# Formulation and Evaluation of Insulin-Loaded Sodium-Alginate Microparticles for Oral Administration

**DOI:** 10.3390/pharmaceutics16010046

**Published:** 2023-12-28

**Authors:** Ildikó Bácskay, Boglárka Papp, Péter Pártos, István Budai, Ágota Pető, Pálma Fehér, Zoltán Ujhelyi, Dóra Kósa

**Affiliations:** 1 Department of Pharmaceutical Technology, Faculty of Pharmacy, University of Debrecen, Nagyerdei Körút 98, 4032 Debrecen, Hungaryfeher.palma@pharm.unideb.hu (P.F.); ujhelyi.zoltan@pharm.unideb.hu (Z.U.); 2 Institute of Healthcare Industry, University of Debrecen, Nagyerdei Körút 98, 4032 Debrecen, Hungary; 3 Faculty of Engineering, University of Debrecen, Ótemető Utca 2-4, 4028 Debrecen, Hungary; budai.istvan@eng.unideb.hu

**Keywords:** microbead, oral bioavailability, absorption enhancement, Labrasol ALF, Labrafil M 2125 CS, Caco-2 cells

## Abstract

The development of oral insulin drug delivery systems is still an ongoing challenge for pharmaceutical technology researchers, as the formulation process has to overcome a number of obstacles due to the adverse characteristics of peptides. The aim of this study was to formulate different sodium-alginate microparticles as a possible method for oral insulin administration. In our previous studies, the method has been successfully optimized using a small model peptide. The incorporation of insulin into alginate carriers containing nonionic surfactants has not been described yet. In order to enhance the absorption of insulin through biological barriers, Labrasol ALF and Labrafil M 2125 CS were selected as permeation-enhancing excipients. They were applied at a concentration of 0.10% (*v*/*v*%), along with various combinations of the two, to increase oral bioavailability. Encapsulation efficiency showed sufficient drug incorporation, as it resulted in over 80% in each composition. In vitro dissolution and enzymatic stability test results proved that, as a pH-responsive polymer, alginate bead swelling and drug release occur at higher pH, thus protecting insulin against the harsh environment of the gastrointestinal tract. The remaining insulin content was 66% due to SIF degradation after 120 min. Permeability experiments revealed the impact of permeation enhancers and natural polymers on drug absorption, as they enhanced drug transport significantly through Caco-2 cells in the case of alginate microparticle formulations, as opposed to the control insulin solution. These results suggest that these formulations are able to improve the oral bioavailability of insulin.

## 1. Introduction

According to the World Health Organization, 422 million people worldwide have diabetes, and 1.5 million deaths are directly attributed to diabetes each year. Both the number of cases and the prevalence of diabetes have been constantly increasing over the past decades. The most effective therapy for patients living with diabetes mellitus to control high blood sugar level is insulin administration [1]. However, insulin administration is available almost exclusively in injectable form, despite the fact that it has several drawbacks [2]. Continuous injections are painful, inconvenient, and lead to low patient compliance [3,4]. In the long-term, access to an affordable and more comfortable treatment would be crucial. However, the development of oral insulin drug delivery systems is still an ongoing challenge for pharmaceutical technology researchers, as the formulation process has to overcome a number of obstacles due to the adverse characteristics of peptide-type drugs [5]. The frequent enzymatic degradation in the gastrointestinal tract, the low permeability and the physical barriers, all make the formulation of oral dosage forms difficult [6]. To overcome the abovementioned limiting factors associated with oral insulin delivery, several strategies have been investigated in the last decades [7,8]. Orally administered formulations must meet the following requirements: they must protect the drug from the harsh acidic conditions and degrading action of pepsin in the stomach, and several other proteolytic enzymes in the intestinal lumen [9]. Chemical modification of the peptide and enzyme inhibitors helps address this challenge [10]. In order to reach the site of action and achieve the required pharmacological effect when administered orally, we have to face the biological membranes as well. Absorption enhancers temporarily interrupt membrane integrity in order to improve drug permeation through the intestinal and basal membranes [11]. For this purpose, non-ionic surfactants are commonly used, as they are relatively less toxic than other excipients [12]. For many years, extensive research has been conducted to investigate innovative methods for administering insulin, including approaches like micro- and nanoparticles. Among the many options, polymer-based delivery systems gained more focus due to their easy formulation process. Both natural and synthetic polymers have been used to formulate polymer-based delivery systems for oral insulin administration [13,14]. However, natural polymers have been of greater interest due to their high biocompatibility and low toxicity [15]. The two most investigated natural polymers are alginate and chitosan. In recent years, several studies have investigated different alginate-based insulin formulations that seem promising for increasing the oral bioavailability of insulin [16,17,18,19]. The great benefit of sodium-alginate lies in its status a non-toxic, biocompatible and biodegradable polysaccharide. The mucoadhesive property of sodium alginate increases the absorption of oral insulin, making it a potential excipient for designing drug delivery dosage forms [20]. In the presence of divalent cations, such as calcium, sodium alginate crosslinks and forms a polymer matrix that controls drug release at specific pH [21]. Lower pH inhibits the release of drugs, as sodium alginate microparticles are stable in acidic conditions, while higher pH promotes the disintegration of the microsphere structure, thus increasing release rate [22]. The formulation of insulin-loaded calcium cross-linked sodium-alginate microparticles containing different non-ionic surfactants has not been particularly investigated.

Incorporation of insulin into alginate carriers containing nonionic surfactants has not been described yet. For this purpose, we intended to formulate and investigate different sodium-alginate formulations containing two polyoxylglyceride-type permeation-enhancing agents. Labrasol ALF and Labrafil M 2125 CS were selected in order to improve the absorption of the active ingredient through the intestinal mucosa [23]. Microbeads contained these excipients at a concentration of 0.10% (*v*/*v*%), as well as combinations of them. The efficacy and safety of these excipients have been investigated in several studies [24,25,26,27]. Cross-linking of alginate with calcium occurred with the help of a semi-automated instrument, making the formulation process much easier and faster, based on our previous experiments [28]. A number of in vitro investigations were carried out to characterize the microbeads and investigate the protective effect of the polymer in simulated gastrointestinal conditions. Since safety is an essential aspect of pharmaceutical developments, the biological properties of the excipients and compositions were evaluated as well [29]. Overall, the aim of our research was to formulate suitable delivery systems for oral insulin delivery with improved bioavailability.

## 2. Materials and Methods

### 2.1. Materials

Human recombinant insulin, pepsin (≥400 unit/mg protein), and pancreatin (≥3× USP specifications) were obtained from Sigma-Aldrich (St. Louis, MO, USA). Sodium alginate was purchased from BÜCHI Labortechnik AG (Flawil, Switzerland). Calcium chloride dihydrate was ordered from VWR International (Debrecen, Hungary). Labrasol ALF (Caprylocaproyl Prolyoxyl-8-glycerides) and Labrafil M2125 CS (Linoleoyl Polyoxyl-6 glycerides) were purchased from Gattefossé (Saint-Priest, France). The Caco-2 cell line was obtained from the European Collection of Cell Cultures (ECACC, Public Health England, Salisbury, UK). MTT dye (3-(4,5-Dimethylthiazol-2-yl)-2,5-diphenyltetrazolium bromide), phosphate buffered saline (PBS) buffer solution, Dulbecco’s Modified Eagle’s Medium (DMEM), heat-inactivated fetal bovine serum (FBS), L-glutamine, non-essential amino acids solution, and penicillin-streptomycin solution were obtained from Sigma-Aldrich (St. Louis, MO, USA). TrypLE™ Express Enzyme (no phenol red) and Pierce™ Detergent Compatible Bradford Assay Kit were ordered from Thermo Fisher Scientific (Waltham, MA, USA). 

### 2.2. Methods

#### 2.2.1. Formulation of Insulin-Loaded Sodium-Alginate Microparticles

Insulin-loaded alginate microparticles were formulated using the controlled polymerization method with the Büchi Encapsulator B-395 Pro apparatus. This process is based on the fact that the controlled, laminar liquid flow is cracked into equally sized beads due to the vibration at the optimal frequency [30]. For the preparation, the peptide was distributed in 20 mL of the polymer 1.50 *w*/*v*% sodium-alginate solution combined with 0.10 *v*/*v*% of penetration enhancers when needed. The polymer–peptide mixture then was loaded into a syringe and forced into the pulsation chamber of the apparatus at the rate of 5.00 mL/min and passed through an electrical field between the nozzle, with an average diameter of 200 µm, and the electrode set at 1000 V, resulting in a surface charge. Due to electrostatic repulsion, the beads dropped into the hardening 100 mM calcium-chloride dihydrate solution separately. Microparticles were then washed with distilled water, filtered with a vacuum pump and dried by lyophilization for 24 h.

#### 2.2.2. Bradford Assay

The insulin content of the formulations was determined with the help of the Pierce™ Detergent Compatible Bradford Assay Kit, which is a rapid and ready-to-use colorimetric method for quantitative analysis of peptides and proteins [31]. Compared to the traditional Bradford reagent, which is incompatible with most detergents, the modified assay reagent is compatible with most of the commonly used detergents and lysis reagents. Similar to the Bradford method, an immediate shift in absorption maximum occurs, from 465 nm to 595 nm, when the dye binds to a protein, resulting in a color change from green to blue [32]. A total of 150 µL of each sample and 150 µL of assay reagent were pipetted into a 96-well plate. For the standard calibration curve, BSA standard solutions were used in predetermined concentrations. In addition, the assay is complete in just 10 min. The assay can be used with samples that contain or do not contain detergent as well.

#### 2.2.3. Encapsulation Efficiency and Drug-Loading Capacity

Insulin encapsulation efficiency was determined indirectly. To define the amount of insulin encapsulated in the beads, 150 µL of undiluted sample was measured from the hardening solution after formulation. Insulin content was calculated via the Bradford Assay. The encapsulation efficiency of insulin was determined by the equation underneath [33]:

(1) $$EE=\frac{Qt-Qh}{Qt}\times 100$$

where *Qt* is the theoretic drug content encapsulated in the beads, and *Qh* is the insulin content that remained in the hardening solution. 

Loading capacity was defined as the difference between the amount of initial insulin and drug left uncapsulated in the hardening solution, expressed as a percentage of the weight of dry microbeads (*Wd*) [17]:

(2) $$LC=\frac{Qt-Qh}{Wd}\times 100$$

#### 2.2.4. Swelling Behavior 

The water absorption capacity of insulin-loaded sodium alginate microbeads was determined gravimetrically. A total of 50 mg of dry beads were placed in 50 mL distilled water at room temperature for 2 h. The swollen beads were then filtered with vacuum filtration. The swelling behavior was calculated from the change in dry and swollen mass of the beads using the following equation [34]:

(3) $$S=\frac{Ws-Wd}{Ws}\times 100$$

where *Ws* is the weight of swollen particles and *Wd* is the weight of dry beads.

#### 2.2.5. Morphology

The morphology, shape, size and surface area of the particles were characterized using a scanning electron microscope (SEM) with the Hitachi Tabletop microscope (TM3030 Plus). For the analysis, samples were attached to a plate covered with double-sided adhesive tape. The accelerating voltage was 5–15 kV during micrography [35].

#### 2.2.6. In Vitro Dissolution

In order to determine drug release from the formulated microbeads, an in vitro dissolution assay was carried out using the USP dissolution apparatus (Erweka DT 800). Dry beads were placed in freshly prepared HCl (pH 1.2) and phosphate (pH 6.8) buffer solution at 37 °C temperature, with the paddle speed set at 100 rpm. A total of 1 mL aliquots from both dissolution media were collected at predetermined time intervals. Fresh-release media were replaced after each sampling. Drug concentration was analyzed using the Bradford assay.

#### 2.2.7. Enzymatic Stability

Enzymatic degradation was performed in the presence of pepsin and pancreatin proteolytic enzymes. Microparticles were placed into 100 mL of simulated gastric fluid (SGF) containing pepsin for 1 h and into simulated intestinal fluid (SIF) containing pancreatin for 2 h, according to the European Pharmacopoeia specifications. The beads were incubated at 37 °C under moderate stirring in both media. The enzymatic reaction was stopped with an equivalent volume of ice-cold reagent (0.1 M NaOH for SGF and 0.1 M HCl for SIF) [36]. The samples were analyzed using the Bradford assay.

#### 2.2.8. Caco-2 Cell Culture

The immortalized human adenocarcinoma Caco-2 cell line was selected for MTT and permeability assays [37]. Cells were maintained through weekly passaging in plastic cell culture flasks in Dulbecco’s Modified Eagel’s medium (DMEM), supplemented with 2 mM of L-glutamine, 100 mg/L gentamycin and 10% heat-inactivated fetal bovine serum. The cells were stored in a 5% CO_2_ cell incubator at 37 °C. 

#### 2.2.9. Caco-2 Cell Viability Assay

The cell viability of immortalized human colon adenocarcinoma Caco-2 cells was evaluated through the MTT assay. The cells were seeded at a density of 10^4^ cells/well on flat bottom 96-well tissue culture plates and allowed to grow for 7 days. For the MTT assay, the DMEM medium was removed, and the cells were treated with the excipients used for the formulation (sodium-alginate, calcium-chloride dihydrate, Labrasol ALF, Labrafil M2125 CS) and with the bead compositions as well. The mitochondrial activity of viable cells was determined after a 3 h incubation with MTT dye. The formed formazan crystal precipitate was dissolved in acidic isopropanol, and absorbance was measured with the FLUOstar OPTIMA Microplate Reader (BMG LABTECH, Offenburg, Germany) at 570 nm against a 690 nm reference. Cell viability was demonstrated as the percentage of the untreated control [38].

#### 2.2.10. Permeability Experiments

For the permeability experiments, the Caco-2 cell line was selected, as it perfectly models the human intestinal absorption of drugs administered orally [39]. Cells were seeded on 24-well ThinCert™ polyester inserts with a 0.40 µm pore size at a density of 4 × 10^4^ cells. Measurements started when the transepithelial electrical resistance (TEER) values reached 800–1000 Ω × cm^2^ in each insert [40]. The DMEM culture medium was replaced with test solutions in the apical chamber, and phosphate buffer solution was added to the basal chamber. In permeability tests, all the four compositions have been studied. For this experiment the same amount of dry microbead samples were dissolved in PBS buffer for 120 min. As control, insulin solution was used. After 120 min, samples were collected from the basolateral compartment to determine the permeated amount of insulin. The samples were analyzed using the Bradford assay. 

#### 2.2.11. Transepithelial Electrical Resistance Measurements

To follow membrane function and integrity during the permeability experiments, transepithelial electrical resistance (TEER) was measured with Millipore Millicell-ERS 00001 equipment [41]. As a follow-up, measurements were carried out 12 h after incubation to study cell membrane recovery.

#### 2.2.12. Statistical Analysis

Data were analyzed using the GraphPad Prism 8 and herein presented as means ± SD. The results were compared using one-way ANOVA and repeated-measures ANOVA followed by Tukey’s or Dunnett’s post hoc testing. Difference of means was regarded as significant in case of *p* < 0.05 and signed with asterisks. All experiments were carried out in quintuplicates and repeated at least five times.

## 3. Results

### 3.1. Formulation of Insulin-Loaded Sodium-Alginate Microparticles

Insulin was encapsulated in different alginate formulations containing penetration enhancer excipients in a concentration of 0.1% (*v*/*v*%). The selected compositions are described in Table 1.

### 3.2. Encapsulation Efficiency and Drug-Loading Capacity

**Figure 1:**
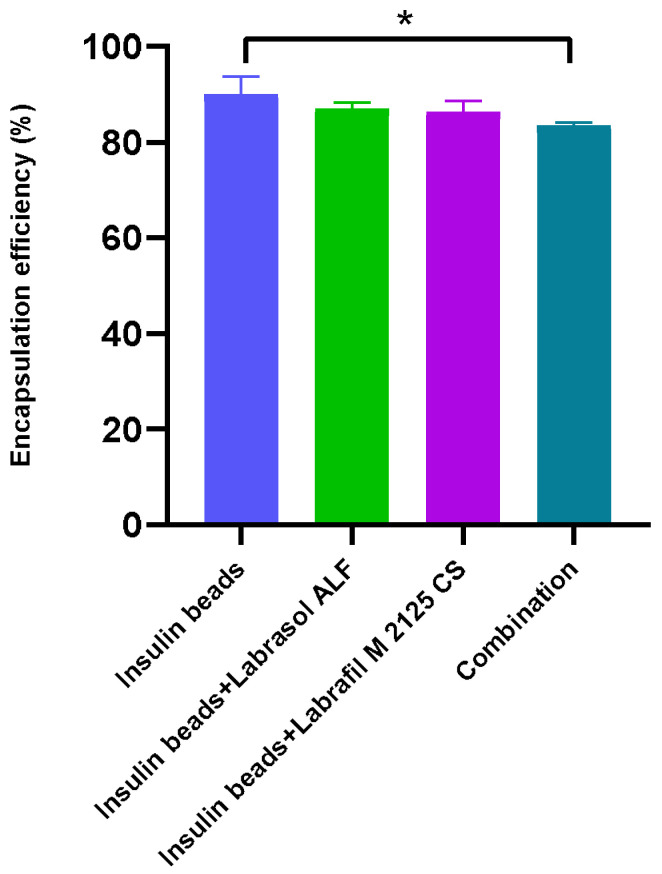
Encapsulation efficiency of insulin. EE measurements showed at least 80% in each case. A significant difference was observed between the formulations without surfactants and those that contained the combination of them. Each data point represents the mean ± SD, *n* = 5. One-way ANOVA with Tukey’s post hoc test was performed to compare the different groups. Significant differences are marked with asterisks. * Indicates statistically significant differences at *p* < 0.05.

The encapsulation efficiency of insulin in the beads was over 80% in each case. A significant difference was evaluated between the EE of the compositions with both excipients. The lowest value was observed in the case of insulin beads containing both penetration enhancers, as the surfactant content was twice as high in those particles. The insulin beads supplemented with only one of the excipients (Labrasol ALF or Labrafil M2125 CS) showed almost the same EE. The drug-loading capacity results were between 1.28 and 1.49%. The results are presented in Figure 1 and Table 2.

### 3.3. Swelling Behavior 

**Figure 2:**
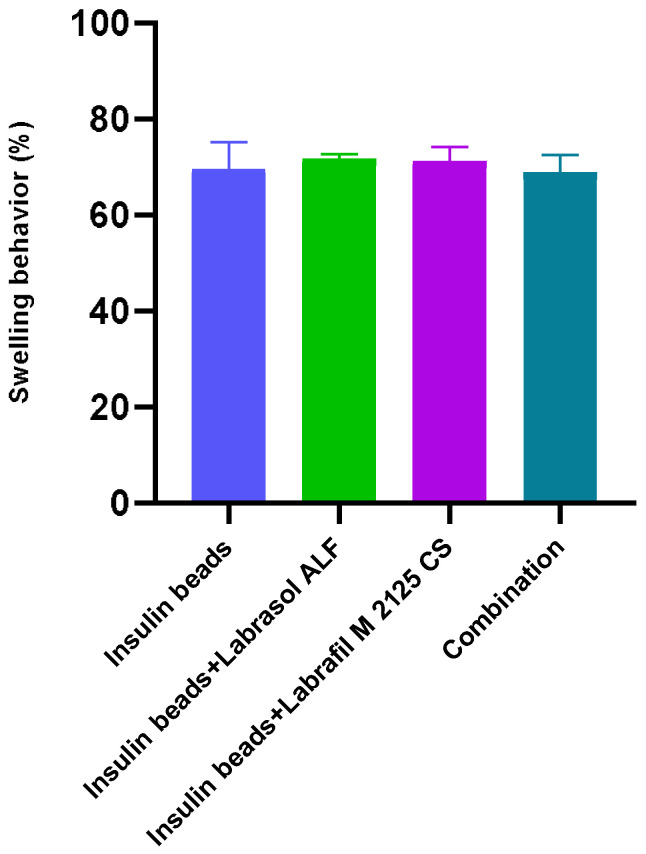
Swelling behavior of the formulated beads after 2 h. Excipient content did not affect equilibrium water uptake significantly. Each data point represents the mean ± SD, *n* = 5. One-way ANOVA was carried out to compare the groups. No significant difference was observed in swelling behavior of the beads.

The swelling behavior of the beads formulated with a 200 µm nozzle was approximately 70%. Bead swelling was 3.5–4 times their dry mass, regardless of the formulation and excipient content. It has been shown that bead swelling is not affected by the excipients. The results of swelling capacity are presented in Figure 2.

### 3.4. Morphology

**Figure 3:**
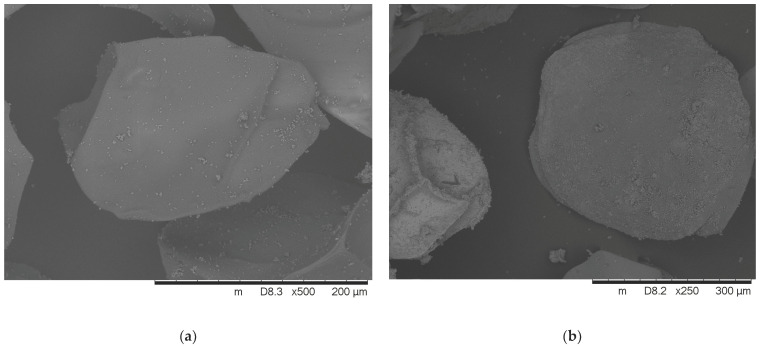

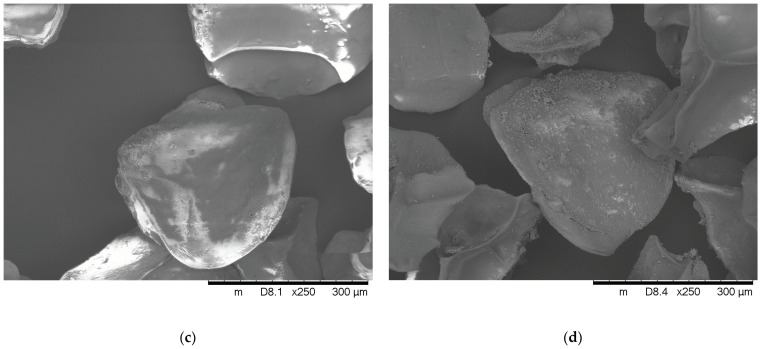
SEM micrographs of insulin-loaded alginate beads: (**a**) insulin beads; (**b**) insulin beads containing Labrasol ALF; (**c**) insulin beads containing Labrafil M2125 CS; (**d**) insulin beads containing both excipients.

The morphology of the lyophilized insulin-loaded alginate microparticles is depicted in Figure 3. The SEM images of dry microspheres present flattened sphere-shaped beads with squashes due to the drying process. Small calcium-chloride crystals can be observed on the bead surface as well. SEM analysis also confirmed that the diameter of the microbeads is close to 200 µm. The average diameter of the formulated microparticles is presented in Table 3. 

### 3.5. In Vitro Dissolution

**Figure 4:**
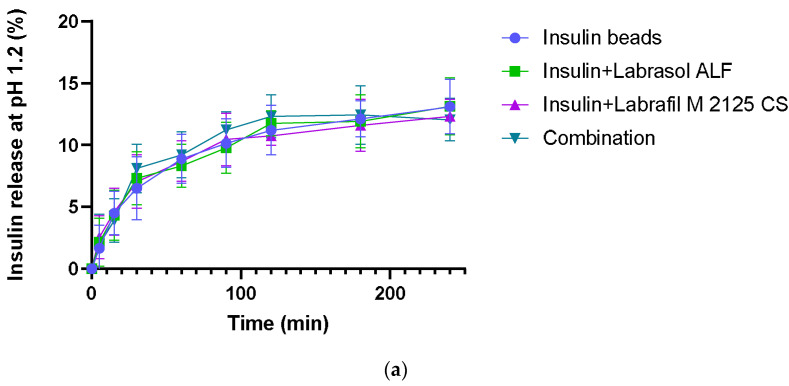

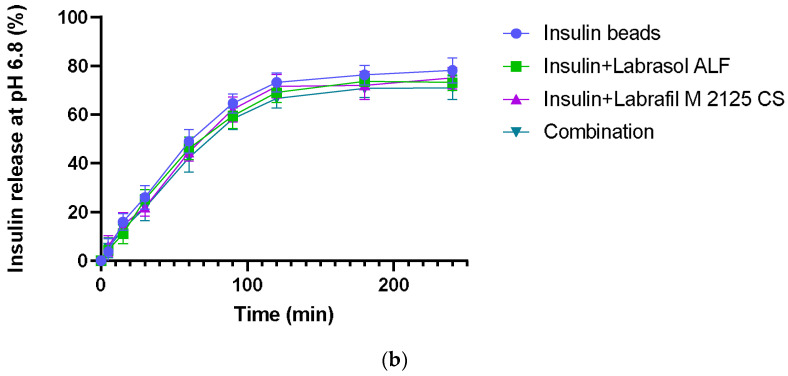
In vitro dissolution of insulin: (**a**) in HCl (pH = 1.2); (**b**) in phosphate buffer solution (pH = 6.8). Each data point represents the mean ± SD, *n* = 5.

In vitro dissolution experiments were carried out in HCl (pH 1.2) and phosphate buffer solution (pH = 6.80). Insulin release at pH 1.2 was very slow, with less than 13% of drug content released within 120 min. At higher pH, in the first 2 h, a burst release of insulin was observed, where insulin release from the microparticles was over 66%. After 2 h, the insulin release rate was much lower. The excipient content did not affect insulin dissolution significantly. Figure 4 shows the percentage of released drug from sodium-alginate beads by time.

### 3.6. Enzymatic Stability

**Figure 5:**
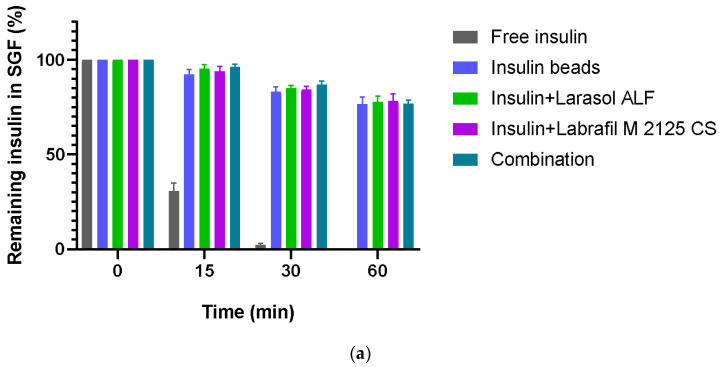

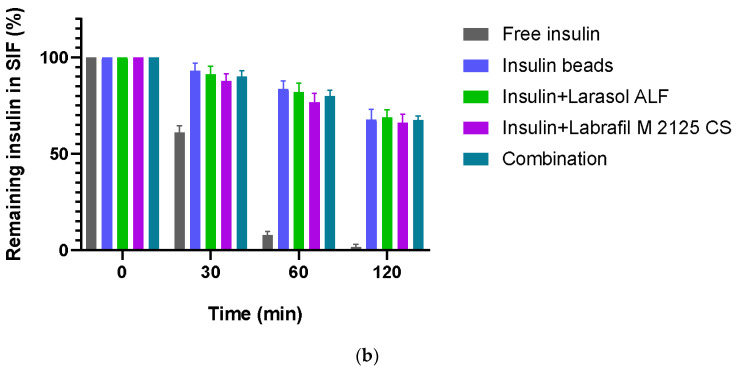
Enzymatic stability of insulin encapsulated in sodium-alginate microparticles: (**a**) in simulated gastric fluid containing pepsin; (**b**) in simulated intestinal fluid containing pancreatin. The control was free insulin. Each data point represents the mean ± SD, *n* = 5. Compared to free insulin, all four alginate formulations significantly protected insulin from enzymatic degradation.

In simulated gastric fluid, less than 2.50% of insulin remained after a 30 min incubation in the case of the non-formulated insulin samples, and free insulin was completely degraded within 1 h of incubation. In simulated intestinal conditions, less than 2% of active insulin was measured after 2 h incubation. According to the results, our formulations were able to protect insulin against the enzymatic conditions of GIT, as at least 80% of insulin remained protected from SGF degradation after 60 min and 66% from SIF degradation after 120 min. Figure 5 represents the results of the study.

### 3.7. Caco-2 Cell Viability Assay

**Figure 6:**
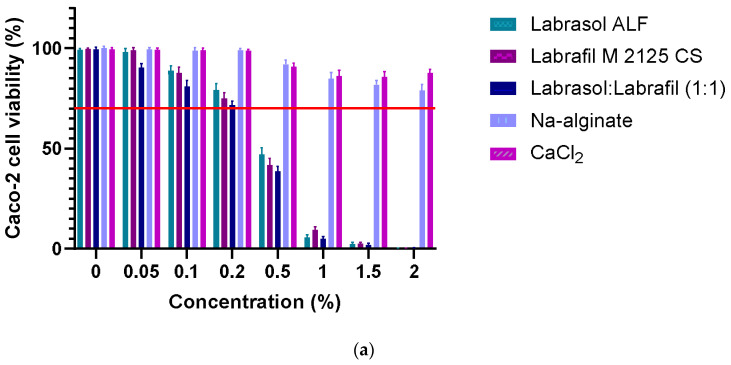

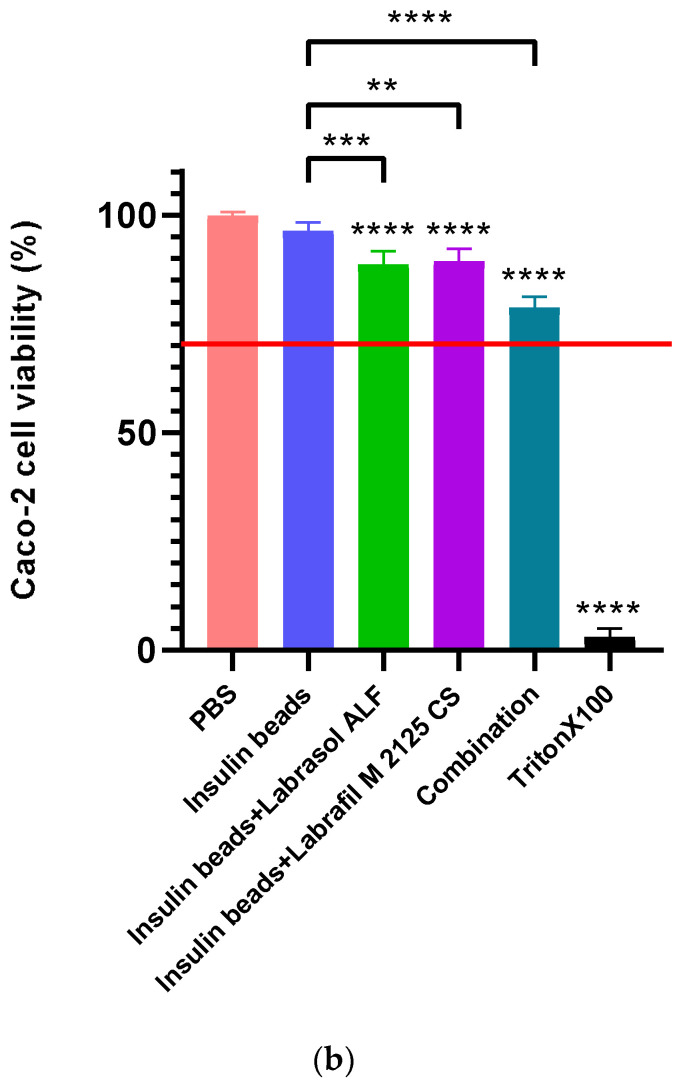
Results of MTT cell viability measurements of: (**a**) the applied excipients; (**b**) the formulated sodium-alginate microbeads containing insulin. Neither the selected excipients nor the bead formulations showed toxicity at the applied concentration according to ISO 10993-5 recommendation, while Labrasol ALF and Labrafil M 2125 CS seemed to be toxic at higher concentrations. The positive control was Triton X-100, the negative control was a phosphate-buffered solution (PBS). Each data point represents the mean ± SD, *n* = 5. One-way ANOVA with Tukey’s post hoc test was carried out to compare the groups. Significant differences are marked with asterisks. The asterisks **, *** and **** indicate statistically significant differences at *p* < 0.01, *p* < 0.001 and *p* < 0.0001.

The Caco-2 cell viability assay results demonstrate that the selected excipients are all safe at the applied 0.10% (*v*/*v*%) concentration. The bead-forming polymer and the hardening solution did not seem to be toxic, even at higher concentrations, in contrast with the permeation enhancers. As for the formulations, the 0.10% (*v*/*v*%) penetration enhancer content did not result in cell damage; all four formulations proved to be safe under in vitro conditions. Overall, cell viability was over 70% in each case, in line with the ISO 10993-5 [42] recommendation. Figure 6a demonstrates the results of the MTT assay regarding excipients, while Figure 6b represents the results of the formulated compositions.

### 3.8. Permeability Experiments

**Figure 7:**
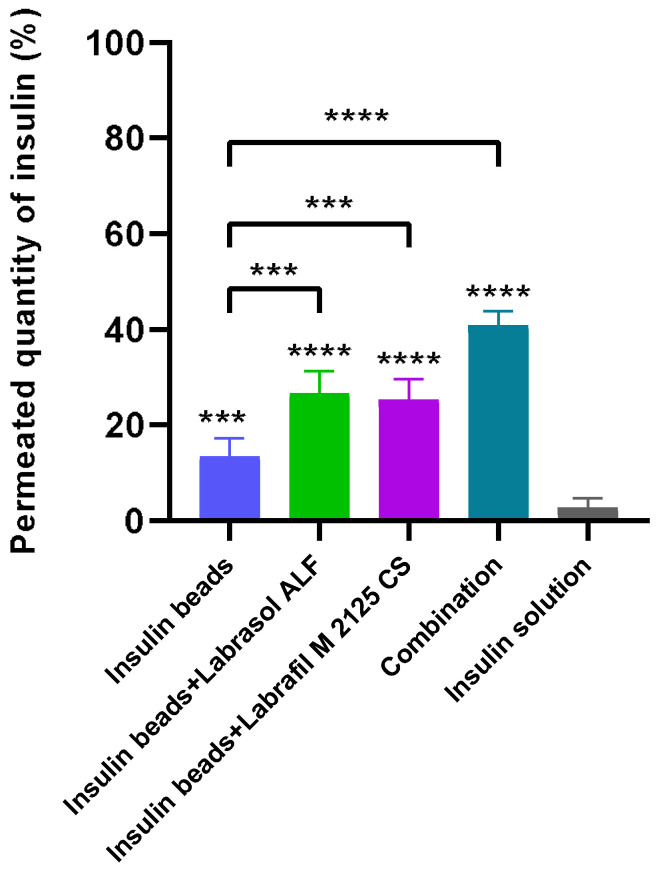
Permeated quantity of insulin via Caco-2 cell monolayer. Insulin solution was applied as control. The results demonstrate an increased peptide permeability in case of formulations containing penetration enhancer excipients. Each data point represents the mean ± SD, *n* = 5. To compare the groups, one-way ANOVA with Dunnett’s multiple comparisons test was performed. Significant differences are marked with asterisks. The asterisks *** and **** indicate statistically significant differences at *p* < 0.001 and *p* < 0.0001.

Figure 7 demonstrates the results of insulin permeability experiments. The permeability of encapsulated insulin was significantly higher than that of the control insulin solution. In the case of the formulations containing penetration enhancers, increased drug permeability was measured, suggesting the opening of tight junctions. The best API permeability was reached with beads containing both penetration enhancer excipients. 

### 3.9. Transepithelial Electrical Resistance Measurements

**Figure 8:**
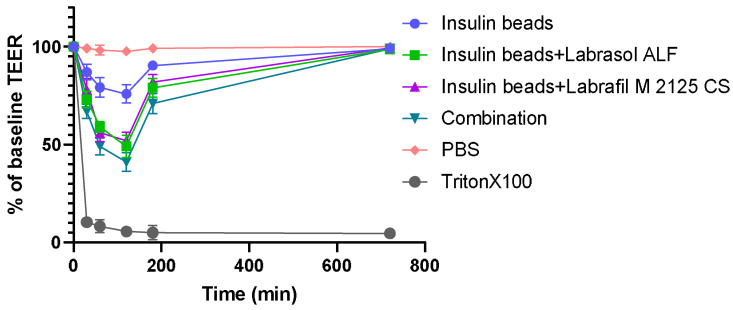
Transepithelial electrical resistance of Caco-2 cells during permeability assessment and 12 h after treatment. The follow-up measurements confirmed that TEER values started to increase after the treatment. At the end of the experiment TEER was above 90% of the baseline. Each data point represents the mean ± SD, *n* = 5.

The permeability test started when the Caco-2 monolayer reached high (800 Ω × cm^2^) TEER values. During the drug permeability investigation, the membrane integrity of Caco-2 cells was monitored through TEER measurements. After 30 min, the formulations started to cause a decrease in TEER values, suggesting the opening of tight junctions. Follow-up measurements confirmed that the TEER values started to increase after the treatment. At the end of the experiment, TEER was above 90% of the baseline. Figure 8 presents the results of TEER measurements.

## 4. Discussion

The oral bioavailability of hydrophilic macromolecular drugs, such as peptides and proteins, is extremely low due to their low stability and poor membrane permeability in the gastrointestinal tract. Natural polysaccharides have been widely investigated as potential delivery systems to improve the oral bioavailability of peptides and proteins in the last decades, still remaining a subject of great interest [43]. The objective of this investigation was to formulate optimal delivery systems for oral insulin administration. For this purpose, sodium-alginate was selected as a drug carrier polymer due to its beneficial properties in combination with two non-ionic surfactants as permeation enhancers. Insulin-loaded alginate microbeads were prepared using a controlled-gelification method with the help of the Büchi Encapsulator B-395 Pro apparatus. 

The encapsulation efficiency of insulin exceeded 80% in each composition. The EE was significantly lower in the case of beads containing the combination of surfactants. Higher surfactant content changes the wetting angle of sodium-alginate solution when it falls into the calcium-chloride solution, resulting in an increase of surface area [44,45,46]. Water uptake was also investigated by swelling the beads in distilled water, as it influences drug release and further application as well. Swelling behavior resulted in at least 70% and was not affected by the excipients.

The performed scanning electron microscopy images confirmed that the morphology and shape of the beads are rather a flattened sphere, which is in contrast with the expected spherical morphology. This phenomenon is caused by the abovementioned increase in the wetting angle caused by the permeation enhancers. Squashes and tiny calcium crystals were also observed on the surface due to lyophilization, as the surface of the beads remains wet after vacuum filtration [34]. The average particle size seemed to be close to the theoretical 200 µm; according to the operation manual of the Büchi apparatus, the diameter of calcium-alginate beads is usually bigger than the nozzle diameter. 

Insulin release from microbeads was investigated at pH 1.2 and 6.8 as well, and showed pH dependence, as expected [47]. Being a pH-responsive polymer, the relatively intact microstructure of alginate in acidic conditions, due to alginic acid, resulted in a slow- release rate at pH 1.2. In contrast, at higher pH, alginate forms a soluble salt, causing matrix swelling and disintegration, leading to higher drug release [48]. The stability of insulin in SGF containing pepsin and in SIF containing pancreatin was also studied. Compared to free insulin, a significant amount of API remained intact in both media after incubation time, suggesting the protective effect of the alginate matrix. A relatively higher degradation was observed in SIF than in SGF, which might be explained by bead swelling and drug release at higher pH. 

In order to improve the intestinal absorption of insulin, different non-ionic surfactants and their combination were incorporated as absorption enhancers [49,50]. These excipients have the ability to modulate tight-junctions reversibly, thus increasing paracellular transport and intestinal permeability [51]. Our results have demonstrated the beneficial effects of surfactants in increasing permeability, as significantly more insulin permeated through Caco-2 cells seeded on the artificial membrane in the case of sodium-alginate microparticle formulations (*p* < 0.0001). Among the four compositions, those containing permeation enhancers supported significantly better API transport than insulin beads (*p* < 0.001). Applying the combination of Labrasol ALF and Labrafil M 2125 CS reached the highest permeated insulin quantity indicating improved bioavailability (*p* < 0.0001). Furthermore, alginate and other natural polymers have the ability to open epithelial cell tight junctions temporary as well, thus modulating the paracellular permeability of cell monolayers [52]. The significant difference between the control insulin solution and the insulin beads indicates that microparticulate systems are able to exert this effect. The TEER measurements performed confirmed our results, as the decreased TEER values during incubation suggest the modulation of cell integrity, while follow-up measurements confirmed that neither alginate nor the applied surfactants altered tight junctions irreversibly. 

The cytotoxicity of the applied polymer solution, hardening solution and surfactants has been evaluated on Caco-2 cells using the well-known MTT assay. It is still one of the most popular in vitro methods to investigate cell viability [53]. Our results confirmed that neither the selected excipients nor the bead formulations showed toxicity at the applied concentration. Since safety is important in pharmaceutical dosage forms, the MTT assay was performed for the microbead formulations as well. This analysis proved their safety for cells under in vitro conditions. 

Our results suggest that the incorporation of non-ionic surfactants with calcium cross-linked alginate microparticles is a promising option to improve the oral bioavailability of insulin. The carefully selected excipients and alginate are both able to enhance the intestinal absorption of the active substance as well as protecting it from the enzymatic degradation of the gastrointestinal tract. 

## 5. Conclusions

The aim of the study was to formulate stable oral delivery systems that allow for enhanced in vitro drug release and intestinal absorption of insulin and to create a well-tolerated drug formulation that provides a high degree of protection against drug degradation. In our previous research, we successfully formulated alginate microparticles containing surfactants with a small model peptide as promising delivery systems for peptide-type active substances. In order to achieve better oral bioavailability, we selected other excipients, as well as their combination, and increased the concentrations tenfold. According to the results, our formulations were still safe under in vitro conditions. Further in vivo studies could demonstrate the importance of these formulations in insulin therapy. Formulations developed with such an approach would increase patient compliance with insulin therapy, thus playing an important role in the treatment of a leading disease.

## Figures and Tables

**Table 1:** Composition of the selected alginate formulations.

Composition	Sodium-Alginate Solution	Labrasol ALF	Labrafil M2125 CS
Insulin beads	20 mL	-	-
Insulin beads + Labrasol ALF	20 mL	0.1% (*v*/*v*%)	-
Insulin beads + Labrafil M2125 CS	20 mL	-	0.1% (*v*/*v*%)
Combination	20 mL	0.1% *v*/*v*%	0.1% (*v*/*v*%)

**Table 2:** Loading capacity of the formulated insulin-alginate compositions.

Composition	LC (±SD; %)
Insulin beads	1.45 ± 0.15
Insulin beads + Labrasol ALF	1.49 ± 0.14
Insulin beads + Labrafil M2125 CS	1.34 ± 0.03
Combination	1.28 ± 0.09

**Table 3:** Average diameter and polydispersity index (PDI) of the formulated microparticles.

Composition	Diameter of Lyophilized Microspheres (±SD; µm)
Insulin beads	277.8 ± 11.95
Insulin beads + Labrasol ALF	292.9 ± 9.56
Insulin beads + Labrafil M2125 CS	296.3 ± 10.19
Combination	298.4 ± 8.21

## Data Availability

The data that support the findings of this study are available from the corresponding author (kosa.dora@pharm.unideb.hu) with the permission of the head of the department, upon reasonable request.
